# Motion Illusions as Environmental Enrichment for Zoo Animals: A Preliminary Investigation on Lions (*Panthera leo*)

**DOI:** 10.3389/fpsyg.2019.02220

**Published:** 2019-10-04

**Authors:** Barbara Regaiolli, Angelo Rizzo, Giorgio Ottolini, Maria Elena Miletto Petrazzini, Caterina Spiezio, Christian Agrillo

**Affiliations:** ^1^Research and Conservation Department, Parco Natura Viva – Garda Zoological Park, Bussolengo, Italy; ^2^School of Biological and Chemical Sciences, Queen Mary University of London, London, United Kingdom; ^3^Department of General Psychology, University of Padova, Padua, Italy

**Keywords:** visual illusions, environmental enrichment, zoo animals, Rotating Snake illusion, animal welfare

## Abstract

Investigating perceptual and cognitive abilities of zoo animals might help to improve their husbandry and enrich their daily life with new stimuli. Developing new environmental enrichment programs and devices is hence necessary to promote species-specific behaviors that need to be maintained in controlled environments. As far as we are aware, no study has ever tested the potential benefits of motion illusions as visual enrichment for zoo animals. Starting from a recent study showing that domestic cats are spontaneously attracted by a well-known motion illusion, the Rotating Snake (RS) illusion, we studied whether this illusion could be used as a visual enrichment for big cats. We observed the spontaneous behavior of three lionesses when three different visual stimuli were placed in their environment: the RS illusion and two control stimuli. The study involved two different periods: the baseline and the RS period, in which the visual stimuli were provided to the lionesses. To assess whether the lionesses were specifically attracted by the RS illusion, we collected data on the number of interactions with the stimuli, as well as on the total time spent interacting with them. To investigate the effect of the illusion on the animals’ welfare, individual and social behaviors were studied, and compared between the two periods. The results showed that two lionesses out of three interacted more with the RS stimulus than with the two control stimuli. The fact that the lionesses seemed to be more inclined to interact with the RS stimulus indirectly suggests the intriguing possibility that they were attracted by the illusory motion. Moreover, behavioral changes between the two periods were reported for one of the lionesses, highlighting a reduction in self-directed behaviors and an increase in attentive behaviors, and suggesting positive welfare implications. Thus, behavioral observations made before and during the presentation of the stimuli showed that our visual enrichment actually provided positive effects in lionesses. These results call for the development of future studies on the use of visual illusions in the enrichment programs of zoo animals.

## Introduction

The perception of the psychophysiological needs of zoo animals has widely changed in the last decades. The increasing number of stimulations and techniques used to provide “environmental enrichment” indeed reflects our higher sensitivity to these perceived needs (Carlstead and Shepherdson, 2000; Makecha and Highfill, 2018). Providing an exhaustive definition of “environmental enrichment” is difficult. To date, nine different types of enrichments can be found in the literature (reviewed by Maple and Perdue, 2013): feeding (e.g., manipulation of food), tactile (e.g., including a water pool to provide tactile stimulation), structural (e.g., changes in the environment, such as introducing a new platform in the enclosure), auditory (e.g., presenting conspecifics’ vocalization in the enclosure), olfactory (e.g., introducing odor from conspecifics or non-conspecifics), visual (e.g., colored objects in the enclosure), social (introducing social companions or individuals of different species), human-animal (e.g., interacting with keepers during feeding time), and cognitive enrichment (e.g., problem-solving tasks to stimulate higher cognitive functions). All of these enrichments are supposed to increase the physical, social, and cognitive complexity of captive environments.

Among visual stimuli, dynamic objects probably represent the most powerful stimuli because motion is known to attract most animals and elicit predator-searching behavior. Unfortunately, setting up artificial contexts in which dynamic objects are presented in the animal enclosure is difficult and costly because it requires installation of some sort of track along which those objects should move repeatedly. Furthermore, if objects move according to a fixed route, they might result in the emergence of stereotypic behaviors of the animals that might start to move forward and backward in the proximity of the area in which the objects are presented. An alternative way to present dynamic objects consists of presenting videotapes to animal zoos. Video presentations were found to be effective in enrichment for chimpanzees (Bloomsmith et al., 1990; Bloomsmith and Lambeth, 2000) and captive macaques (Platt and Novak, 1997). Similarly, videos showing natural landscapes were partially effective in reducing stereotypic behaviors of European starlings kept in captivity (Coulon et al., 2014). Videotapes, however, require the use of monitors and associated electric equipment that must be safely implemented in the naturalistic enclosure, a condition that might represent a problem in natural parks with a limited budget. In this sense, even though dynamic objects seem to present a powerful tool for visual enrichment of zoo animals, for practical reasons, this type of enrichment cannot be easily implemented in natural parks.

With respect to this issue, motion illusions might play an important role. Motion illusions are a sub-category of visual illusions characterized by the perception of motion that is absent in the physical stimulation. Recently it was demonstrated that non-human animals are susceptible to motion illusions: for instance, rhesus monkeys (Agrillo et al., 2015), guppies, and zebrafish (Gori et al., 2014) were shown to perceive the Rotating Snake (RS) illusion. Despite the name, the perception of snakes is limited if not absent. Rather, it is a peripheral drift illusion consisting of the perception of rotational motion for concentric circles in a constant direction. This visual pattern is made by a regular arrangement of colored local elements (Figure 2A): even though the traditional version of the illusion is colored, the illusion is based on a specific achromatic sequence (Kitaoka, 2014): black, dark gray, white, and light gray. In the colored version of the illusion, this gray pattern is hidden in the following sequence: black, blue, white, yellow. The presentation of local information arranged in this order is misperceived in the visual system, leading to a perception of dynamic objects. This seems to be due to the integration of local motion-signal elements in the lateral part of the occipital cortex called the MT complex (Kuriki et al., 2008). Fixational eye movements also seem to be important in eliciting illusory motion. Murakami et al. (2006) and Beer et al. (2008) suggested fixational drifts as the main fixational eye movements underlying illusory motion, whereas Otero-Millan et al. (2012) highlighted the role of transient oculomotor events in initiating illusory motion perception. Billino et al. (2009) found that 84% of human observers experience rotational motion of concentric circles, thus making it one of the most powerful motion illusions in the literature.

A recent online survey suggested that cats might be susceptible to motion illusion, too. In this study, Baath et al. (2014) asked pet owners to present the RS illusion to their cats and then report whether pets showed some sort of behavior that might suggest a perception of motion (for instance, “attacking” the illusory pattern as they were perceiving a living organism in movement): nineteen out of sixty-six respondents declared that their pet reacted to the illusory pattern. Of course, this study was not an empirical laboratory study; animals were observed in non-controlled conditions. Also, data were directly collected by pet owners with their own subjective judgments and feelings that might have interfered with data collection. Above all, even assuming that these data reflect a spontaneous preference of cats to engage in some activities with the RS pattern, it does not necessarily mean that cats do perceive illusory motion *per se*; rather, they might be simply interested in complex visual patterns. That said, this survey study showed that some cats are particularly attracted by the RS pattern, which aligns with the well-known knowledge according to which felines are attracted by moving objects. If cats appear to be interested in interacting with the RS illusion, the possibility exists that motion illusions could represent another type of visual enrichment for big cats kept in captivity. Visual patterns that elicit motion illusions would be less expensive than the equipment necessary to present true dynamic objects and could be easily placed in multiple areas of the enclosure. As far as we are aware, no study has tested the potential benefits of motion illusions in the environment enrichment of zoo animals.

Animal welfare has been defined as “the state of an animal as regards its attempts to cope with its environment” (Broom, 1986; Hill and Broom, 2009) and can be assessed scientifically by investigating how animals try and achieve to do so (Hill and Broom, 2009). Welfare can vary on a continuum from very good to very poor (Broom, 1988). On the contrary, the presence of species-specific behaviors in zoo animals has been considered a valuable measure of psychological and physiological well-being, with behavioral similarities between captive and wild animals indicating a positive welfare state (Hill and Broom, 2009; Hosey et al., 2013). On the other hand, abnormal and stress-related behaviors such as over-grooming might indicate poor welfare conditions and high individual stress levels (Dawkins, 1990; Lutz et al., 2003; Jacobson et al., 2016). Carnivores in controlled environments are known to be inactive and prone to exhibit abnormal behaviors (Powell, 1995). However, different types of environmental enrichments have been found to increase activity levels, promote functional and natural behaviors, and reduce abnormal behaviors in different species of felids (*e.g*., *Panthera tigris* and *Panthera leo*: Powell, 1995; Bashaw et al., 2003; Van Metter et al., 2008; *Leopardus geoffroyi, L. tigrinus* and *L. wiedii*: Resende et al., 2009).

In the present study, we investigated the effects of motion illusions as a visual enrichment for big cats. In many respects, human and feline vision are comparable. These similarities encompass stereopsis (Fox and Blake, 1971), rod/cone discontinuity during dark adaptation, the Purkinje shift (La Motte and Brown, 1970), a 5-octave range of spatial frequencies (Blake et al., 1974), and a trade-off in sensitivity between spatial and temporal resolution (Blake and Camisa, 1977). Concerning color discrimination, there is a debate as to whether primates and felines experience a comparable color perception. It has been argued that felines have a dichromatic spectral sensitivity that closely resembles red-green color-blindness in humans (Clark and Clark, 2016). However, the three-cone cat retina described by Ring et al. (1977) resembles the extramacular retina found in macaques, suggesting thricromatic vision in felines too.

To achieve our goal, we presented the RS illusion (physically static stimulus that appears to be dynamic to human observers) and two control stimuli (physically static stimuli that also appear to be static) to three adult lionesses. Because the illusory motion elicited by this pattern is not related to color but to a specific gray sequence, any potential difference in color perception between primates and felines is not expected to alter the perception of motion in lionesses. To assess whether lionesses are naturally attracted by the stimulus associated with illusory motion, we recorded the number of times they approached the stimuli and the time spent interacting with the three stimuli. To ensure that such interaction led to concrete positive benefits in the animal welfare, individual and social behaviors of the lionesses were observed before and during the presentation of our stimuli using a continuous focal animal sampling method.

## Materials and Methods

### Subjects

**FIGURE 1:**
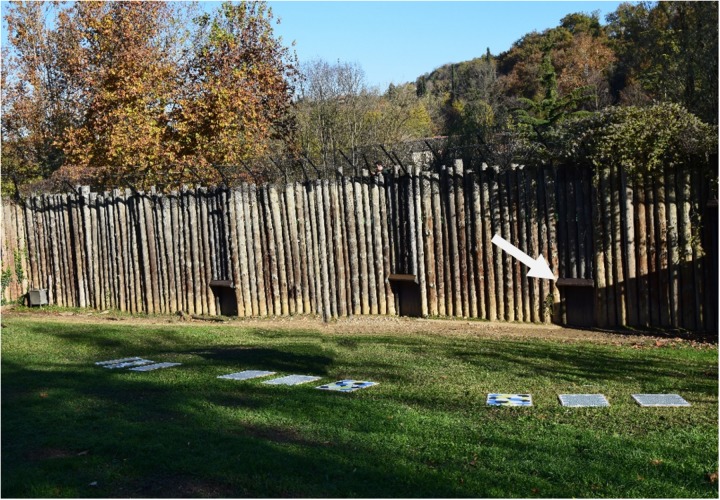
Arrangement of the three triplets of stimuli in the outdoor area of the enclosure. The three triplets were arranged in a semicircle around the guillotine door through which the lionesses had access to the outdoor area (indicated by the arrow).

The study was carried out with three lionesses (Safìa, Kianga, and Lubaya) housed at the Parco Natura Viva, a zoological garden in Bussolengo (VR), Italy. The lionesses had been living together in the zoo for 9 years. Safìa, the dominant female, was a white lioness (ssp. *Panthera leo krugeri*) and was 10 years old, whereas Kianga and Lubaya were sisters and were 9 years old. The lionesses’ enclosure consisted of an outdoor and an indoor area. The outdoor area was 4,359.54 m^2^ and was a grassy exhibit containing vegetation and naturalistic furnishing. The indoor area of the enclosure was composed of different rooms connecting with each other through guillotine doors and was separated from the outdoor area through three guillotine doors, although the lionesses used the same door to move between different areas (Figure 1). The lionesses were fed once a day (6 days a week, with one fasting day) in the indoor area of the enclosure, and no food was provided in the outdoor area. Water was available *ad libitum*. The lionesses were not used to directly interacting with zookeepers and humans in general, as human-animal direct interaction was strictly forbidden in the zoo.

The stimuli of the experimental period were provided to the lionesses as environmental enrichments, and the study subjects were free to decide whether or not to interact with them. The study did not involve any invasive or stressful techniques and was conducted in accordance with the EU Directive 2010/63/EU and the Italian legislative decree 26/2014 for Animal Research.

### Apparatus and Stimuli

**FIGURE 2:**
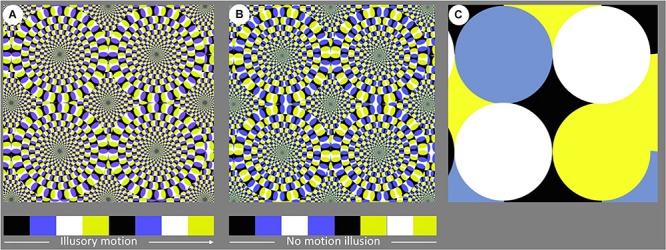
Visual pattern presented within each stimulus: **(A)** Rotating Snake (RS), **(B)** Control 1, and **(C)** Control 2. Stimulus **(A,B)** were identical, with the exception that the sequence of colored segments varied between the two arrays; only the former sequence could elicit the motion illusion in human observers.

The stimuli used in the current study consisted of triplets of wooden panels with illusory and control patterns. In particular, the illusory pattern (RS illusion) (Figure 2A) and two control patterns (C1 and C2) (Figures 2B,C) were printed on PVC sheets (A1, the same size as the panel) and fixed to each wooden panel with screws. Each panel had one PVC sheet, and each triplet of panels contained one RS, one Control 1 and one Control 2. The patterns on the three PVC sheets within each triplet were:

#### The Rotating Snake (RS)

The Rotating Snake was the illusory pattern used in this study (Figure 2A). The alternation of black, blue, white, and yellow segments causes an illusory sense of movement in human observers.

#### Control 1 (C1)

This stimulus, previously adopted in studies on human (Kuriki et al., 2008), and non-human animals (Gori et al., 2014; Agrillo et al., 2015), does not evoke any motion perception, even though the overall configuration is identical to that of the illusory pattern (Figure 2B). Because the order of the colored segments is reversed between adjacent units, the local motion signal is nulled. This stimulus represents a powerful tool to assess whether lions are attracted by the apparent movement of the RS or simply by a complex visual pattern in which different colors alternated. If the former were true, we would expect a preference for exploring the RS stimulus; if the latter were true, no difference would be reported between the two stimuli.

#### Control 2 (C2)

This is the control pattern that differs from the first two visual stimuli (Figure 2C). It consists of overlapping circles, but the overall stimulus is extremely less complex than RS and C1. In this sense, this stimulus acted as a check to verify whether simple visual stimuli could attract the lions. No illusory motion can be perceived with this pattern by human observers.

### Procedure and Data Collection

#### Assessing the Preference for RS Array

To assess whether the lionesses exhibited a spontaneous preference for interacting with the visual pattern that elicits a vivid perception of motion in human observers, we collected data on the interaction with the PVC panels during the RS period, in which the stimuli were provided. Nine sessions were carried out over approximately 1 month. Within each session, to avoid competition, lionesses were provided with three triplets, each with the RS, C1, and C2. Within each triplet, the three panels were placed approximately 30 cm from each other. The disposition of the visual stimuli varied within triplets and over the study sessions and was defined based on a pseudo-random schedule. The three triplets were placed at approximately 1.5 m (based on the lioness length of head and body, Haas et al., 2005) from each other and were arranged in a semicircle around the guillotine door linking the outdoor and the indoor areas of the enclosure. The radius of the semicircle was approximately 10 m. The stimuli were placed in the outdoor enclosure in the early morning after cleaning and immediately before the lionesses were moved into the area. When the lionesses passed through the guillotine door and entered the outdoor area of the enclosure, they could immediately see the triplets with the stimuli, because the triplets’ semicircle was on a gentle rising slope (Figure 1).

In the RS period, a GoPro Hero4 camera was positioned at a height of approximately 6 m at the best view from which to video record the interaction of each lioness with the stimuli. The GoPro Hero4 camera was placed every morning before the lionesses entered the outdoor area of the enclosure, where they could find the stimuli. Each video recording started when the lionesses entered the outdoor enclosure (at approximately 9:00 am) and ended when they were called back into the indoor area of the enclosure. On average, each session lasted 5 h. The analysis of the videos allowed us to collect behavioral data of each lioness by using continuous recording with focal animal sampling. For each lioness, the analysis of the videotapes allowed us to collect data on the total number of interactions with RS, C1, and C2 for each subject. Moreover, the duration of each interaction was collected.

#### Effect of Visual Stimuli on the Lionesses’ Behavior

One of the aims of this study was to investigate whether the presence of these visual stimuli affected the behavior and welfare of the study lionesses. To achieve this aim, we collected data on the duration of the individual and social behaviors of the lionesses at baseline, before the provision of the visual stimuli, and during a part of the RS period described above (see section “Assessing the Preference for RS Array”). For each period and each subject, twelve 30-min sessions were done, one in the morning and one in the afternoon, and they were carried out over a 2-week period. Per period, a total of 36 sessions for all lionesses was done, collecting 1,080 min of observation. At baseline, lionesses were observed in the outdoor area of the enclosure using the routine husbandry procedure (sensory enrichment devices impregnated with olfactory stimuli such as spices, perfumes, and herbivore feces). Moreover, in the RS period, the lionesses were observed in the outdoor area of the enclosure where, together with the usual sensory enrichments, the lionesses were provided with the wooden panels with RS, C1, and C2. Durations of the individual and social behaviors of each lioness during the baseline and the RS period were collected using a continuous focal animal sampling method, through the live observation of the subjects. Specifically, data were collected by the same observer, and subjects were observed in a prescribed sequence following a specific design to avoid time-of-day bias. The individual and social behaviors considered in the study are reported in Table 1 and were defined based on preliminary observation of the study subjects as well as on previous literature on the ethogram of lions and other felid species (Powell, 1995; Stanton et al., 2015). The time spent out of sight (hiding or staying away from the visitor/observer area) by the study subjects was also recorded, because in wild animals this condition might indicate a stressful situation or even be informative of chronic stress (Carlstead et al., 1993; Sellinger and Ha, 2005; Davey, 2006; Morgan and Tromborg, 2007; Hosey, 2013).

**TABLE 1:** Behavioral ethogram of the study lionesses.

I**nactivity**	
Inactivity	Laying or crouching with eyes closed

**Activity**	

***Individual behavior***	
Attention	Staring at one area or paying attention to any visual or auditory stimulus
Observing	Looking around calmly
Locomotion	Walking, running or jumping
Maintenance	Yawning, drinking, urinating and defecating
Self-grooming	Licking or scratching of the own body
Scent-marking	Marking substrates or objects in the enclosure by urine-spray (releasing urine backward against a vertical surface or object while standing with tail raised vertically), rolling and rubbing (leaving scents on the substrate or on any object, respectively)
Olfactory exploration	Sniffing the air, an object or the substrate, performing flehmen
Environmental Enrichment	Interacting with an enrichment device by biting, dragging, scratching or carrying it in the mouth
Anticipatory behavior	Moving near the entrance of the indoor area of the enclosure
**Social behavior**	
Affiliative behavior^∗^	Social play (play-fight, chasing, palying together with an enrichment device), putting the front paw or rubbing on a conspecific, social grooming (licking a conspecific or being licked) and paying attention to conspecifics by observing them with interest
Agonistic behavior^∗^	Dominance mount, threat display, aggression
Interspecific behavior	Paying attention to humans such as visitors and zookeepers

**Not observed**	

Out of sight	The animal is not visible from the point of observation (visitor window)

*^∗^Include actions performed or received by the focal subject. The ethogram was made based on preliminary observation of the study lionesses and based on previous literature on lions’ and felids’ behavior (Powell, 1995; Stanton et al., 2015).*

### Data Analysis

Because not all data were normally distributed, the statistical analyses were done using non-parametric statistic tests. In particular, data obtained from the videos collected in the RS period allowed us to determine the preference for different visual patterns, particularly the RS, whereas data collected through the live observation of the subjects at baseline and in the RS period were used to evaluate the effect of the visual stimuli on the welfare of the lionesses. Significance level was set at *p* < 0.05, and all tests were two tailed. Data from the videotapes (RS period) and from the live observation of the lionesses (baseline vs. RS period) were collected by the same observer.

#### Assessing the Preference for RS Array

To assess whether in the RS period lionesses spontaneously preferred to interact with the RS stimulus, we used chi-square tests to establish whether the frequency of interactions was different with the three stimuli; Friedman’s test was used to assess whether the proportion of time spent interacting with the experimental material was statistically different as a function of the type of stimuli. Finally, to verify whether the interest of the lionesses toward the stimuli remained steady over the RS period, a Spearman correlation was run between the number of sessions (from 1 to 9) and both the duration and frequency of interaction with the PVC panels per session.

#### Effects of the Visual Stimuli on Lionesses’ Behavior

Concerning the investigation of the effects of our visual stimuli on lionesses’ behavior, we compared the behavioral data between the baseline and the RS period using a single-case analysis. The Wilcoxon-Mann-Whitney test (software by Marx et al., 2016) was used to compare the durations of individual and social behaviors of each subject between the two periods. In the results, durations are expressed in seconds. For all behavioral categories, medians, interquartile range (IQR), and effect size (*r*) are reported in the manuscript or in figures and tables.

## Results

### Assessing the Preference for RS Array

**FIGURE 3:**
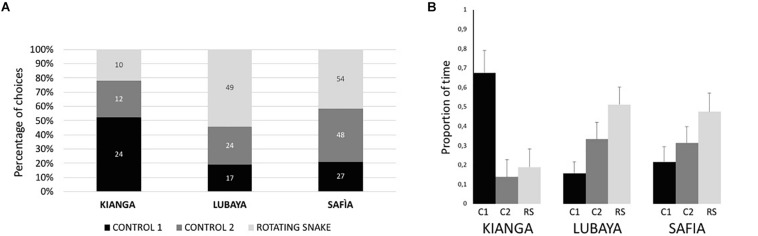
Percentage of times in which lionesses interacted with the three stimuli **(A)** and proportion of time **(B)** spent near the three stimuli (C1, Control 1; C2, Control 2; RS, Rotating Snake). Bars represent the standard error of the mean.

Descriptive data of total interactions and the proportion of time spent near the stimuli are illustrated in Figure 3. Lubaya statistically preferred to approach the RS stimulus [χ2(2) = 18.867, *p* < 0.001, *r* = 0.458]. Friedman’s test showed that the subject spent a different proportion of time near the stimuli, with a larger amount of time near the RS [χ2(2) = 8.026, *p* = 0.018, Kendal’s *W* = 0.174]. Safia statistically preferred to approach the RS stimulus [χ2(2) = 9.349, *p* = 0.009, *r* = 0.269]. However, Friedman’s test showed that the subject did not spend a different proportion of time near the three stimuli [χ2(2) = 2.160, *p* = 0.340, *W* = 0.047]. Kianga statistically preferred to approach Control 1 [χ2(2) = 7.478, *p* = 0.024, *r* = 0.403]. Friedman’s test showed that the subject statistically spent a different proportion of time near the stimuli, with a larger amount of time near Control 1[χ2(2) = 7.682, *p* = 0.021, *W* = 0.295]. The preference of each lioness did not change as a function of time, in terms of both interactions (Spearman’s correlation between total interactions in each trial and experimental sessions, for all subjects, *p* > 0.070) and time spent to explore the stimuli (Spearman’s correlation between proportion of time near the stimuli and experimental sessions, for all subjects *p* > 0.081).

### Effects of the Visual Stimuli on Lionesses’ Behavior

**FIGURE 4:**
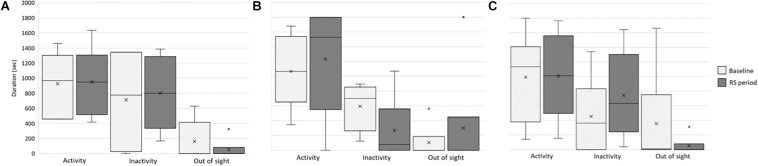
Activity, inactivity, and out-of-sight behavior of the lionesses. Box and whisker plot of the time spent being inactive, active, and out of sight at baseline (light gray) and during the RS period (dark gray) by the study subjects **(A)** Kianga, **(B)** Lubaya, and **(C)** Safia. The horizontal lines within the box indicate the medians; boundaries of the box indicate the first and third quartile. The whiskers extend from the top of the box to the largest data element that is less than or equal to 1.5 times the interquartile range (IQR) and down from the bottom to the smallest data element that is larger than 1.5 times the IQR. Values outside this range are considered outliers and are drawn as points.

To verify whether and how the provision of these visual stimuli impacted the behavior and welfare of the lionesses, we compared activity level and individual and social behaviors between the baseline and RS period. First, we considered whether the stimuli affected the overall activity, inactivity, and out-of-sight condition of each lioness (Figure 4). In all the study subjects, no significant differences were found between the two periods in activity (Kianga: *p* = 0.253, *r* = −0.163; Lubaya: *p* = 0.065, *r* = −0.336; Safia: *p* = 0.381, *r* = −0.091), and out-of-sight condition (Kianga: *p* = 0.149, *r* = 0.303; Lubaya: *p* = 0.608, *r* = −0.083; Safia: *p* = 0.446, *r* = 0.248). In the case of inactivity, Lubaya was more inactive at the baseline than in the second period (*p* = 0.009, *r* = 0.436), whereas no significant differences were found for Kianga (*p* = 0.608, *r* = −0.073) and Safia (*p* = 0.886, *r* = −0.018) (Figure 4).

To gain a better understanding of the effect of the stimuli on the lionesses’ behavior, we considered individual and social behaviors as classes. At baseline, the median (IQR) time spent performing individual behaviors was 708.5 s (438.8) for Kianga, 838 s (883.5) for Lubaya, and 995 s (734) for Safia. In the RS period, the median (IQR) time spent performing individual behaviors was 1343.5 s (798) for Kianga, 1090 s (582.3) for Lubaya, and 1161 s (659.8) for Safia. No significant differences were found for Lubaya (*p* = 0.434, *r* = −0.160) and Safia (*p* = 0.310, *r* = −0.128), whereas Kianga performed more individual behaviors in the RS period than at baseline (*p* = 0.033, *r* = −0.544). On the other hand, at baseline, the median (IQR) time spent performing social behaviors was 548 s (600.3) for Kianga, 192 s (134.8) for Lubaya, and 283 s (372.8) for Safia. In the RS period, the median (IQR) time spent performing social behaviors was 246 s (253.8) for Kianga, 335.5 s (348.8) for Lubaya, and 293 s (406.5) for Safia. Significant differences were found between the two periods. Kianga performed more social behaviors at baseline than in the RS period (*p* = 0.017, *r* = 0.480), and for Lubaya (*p* = 0.024, *r* = −0.288), the opposite pattern was found. No significant difference was reported for Safia (*p* = 1, *r* = 0).

Considering each behavioral category within individual behaviors, we found that Kianga paid significantly more attention and exhibited anticipatory behavior in the RS period than at baseline, whereas the opposite pattern was reported for self-grooming (see Table 2 for median, IQR, time budgets, and *p*-values). Lubaya performed significantly more maintenance in the RS period than at baseline, whereas no other differences were found (see Table 2 for median, IQR, time budgets, *p*-values, and effect size). No significant differences were found between the two periods for Safia (see Table 2 for median, IQR, time budgets, *p*-values, and effect size). Regarding social behaviors, Kianga performed more affiliative behavior in the RS period than at baseline, whereas interspecific behavior was seen significantly more at baseline than in the RS period (see Table 2 for median, IQR, time budgets, and *p*-values). No significant differences were found in any behavioral category for Lubaya and Safia (see Table 2 for median, IQR, time budgets, *p*-values, and effect size).

**TABLE 2:** Individual and social behaviors performed by the study lionesses.

	**Attention**	**Self-grooming**	**Env-Enr**	**Locom.**	**Mainten.**	**Scent**	**Olf expl**	**Observing**	**Anticip b.**	**Affil.**	**Agon.**	**Intersp.**
**Kianga**												
Baseline	0 (62.3) 0%	31.5 (98.8) 3%	0 (0) 0%	13 (115.8) 4%	6.5 (16.3), 1%	0 (0) 0%	0 (0) 0%	391 (404.3) 30%	0 (0) 0%	6 (44.8) 1%	0 (0) 0%	467 (604.3) 26%
RS period	119 (119) 7%	0 (10.5) 0%	0 (19.5) 1%	49 (148.3) 5%	8.5 (17) 1%	0 (0) 0%	0 (0) 0%	612 (570.8) 34%	26 (206.3) 16%	75.5 (153.8) 5%	0 (0) 0%	78 (177) 7%
*p*-value	0.005^∗∗^	0.008^∗∗^	0.109	0.722	0.919	1	0.739	0.580	0.014^∗^	0.041^∗^	0.478	0.010^∗^
*r*	−0.433	0.496	−0.327	−0.091	0.027	−0.204	0.109	−0.128	0.449	−0.320	0.274	0.528
**Lubaya**												
Baseline	29 (111.3) 4%	0 (0) 0%	0 (0) 0%	17.5 (186.5) 8%	2 (11) 0%	0 (4.5) 0%	0 (0) 2%	495.5 (605.8) 35%	0 (0) 2%	0 (87.3) 2%	0 (0) 0%	164 (199.3) 12%
RS period	58 (239.3) 8%	0 (30) 1%	0 (3) 0%	58.5 (77.8) 3%	13 (22) 1%	0 (0) 0%	0 (0) 0%	733.5 (570.5) 36%	0 (298.8) 9%	125.5 (221.8) 7%	0 (0) 0%	244.5 (342.8) 15%
*p*-value	0.260	0.590	0.109	0.541	0.036^∗^	0.342	0.739	0.755	0.284	0.053	1	0.542
*r*	−0.281	−0.138	−0.327	0.096	−0.390	0.263	0.109	−0.064	−0.248	−0.381	−0.204	−0.128
**Safìa**												
Baseline	0 (68) 2%	0 (39.8) 2%	0 (0) 0%	56.5 (190.3) 5%	2 (20.8) 2%	0 (3.8) 0%	14 (82.8) 4%	576.5 (626.8) 34%	0 (0) 0%	27 (64) 3%	0 (0) 0%	199 (357) 15%
RS period	67.5 (231) 6%	0 (89) 3%	50 (260.8) 8%	82 (195.8) 6%	36 (78.3) 2%	2.5 (34.3) 1%	0 (9) 1%	442 (530.3)25%	0 (34.5) 4%	58 (196.5)9%	0 (0) 0%	102 (225)10%
*p*-value	0.069	0.672	0.017^∗^	0.447	0.121	0.138	0.175	0.410	0.217	0.126	1	0.272
*r*	−0.351	−0.104	−0.449	−0.127	−0.286	−0.300	0.345	0.368	−0.327	−0.218	–	0.304

*Per subject and per period (baseline and RS period with the visual stimuli), the table reports medians (interquartile range), and mean% time (in seconds) spent performing all the behavioral categories considered in the study (Env-Enr, environmental enrichment; Locom., locomotion; Mainten., maintenance; Scent, scent marking; Olf expl., olfactory exploration; Anticip b., anticipatory behavior; Affil., affiliative behaviors; Agon., agonistic behaviors; Intersp., interspecific behaviors). For each subject, below medians and IQR of the two periods is reported the *p*-value (Wilcoxon-Mann-Whitney test) resulting from the comparison between the baseline and the RS period and the effect size (*r*). ^∗^Significant difference *p* < 0.05. ^∗∗^*p* < 0.01.*

## Discussion

Environmental enrichment has been proven to be a relevant strategy to improve zoo animal welfare. However, the effects of environmental enrichment programs on animal behaviors need to be evaluated to ensure that they positively affect the animals’ well-being, focusing on the response of each individual (Quirke and O’Riordan, 2011). Here, we tested the hypothesis according to which a visual pattern eliciting illusory motion might serve as a useful tool for environmental enrichment of big cats.

Our data show that two lionesses out of three (67%; Lubaya and Safia) preferred to approach the RS more than the other stimuli. This is partially confirmed by the analysis showing that Lubaya also spent more time in correspondence with the RS. This was not observed with Safia, even though the trend was in the same direction. Although limited, our data align with studies on cats (Baath et al., 2014) and encourage future investigation in this direction. It is important to note that the two types of control stimuli differed regarding the complexity of the visual array (that is, Control 1 presented a more complex visual pattern compared to Control 2). The fact that 2/3 lionesses selected the RS more than both control stimuli ensured us that their choice was not based on the mere complexity of the illusory array presented.

Finally, when focusing on the interaction with the visual stimuli by the study lionesses in the RS period, we reported a lack of correlation between the number of sessions of the RS period and the number of interactions or the time spent by the lionesses dealing with the stimuli. These findings suggest that the interest of the study subjects toward the new enrichment devices remained stable over the experimental sessions. Thus, providing this kind of stimulation for nine sessions over approximately 1 month seems to be appropriate to keep the lionesses interested in the stimuli.

Although we cannot directly draw any conclusion on the neural mechanisms involved in the perception of the RS illusion by lionesses, the possibility that they experience illusory motion raises the intriguing question as to whether motion extrapolation in this species is based on similar mechanisms described in humans. As said, illusory motion seems to be generated by the activity of the MT complex in the occipital cortex (Kuriki et al., 2008), as well as the results of fixational eye (Murakami et al., 2006; Beer et al., 2008; Otero-Millan et al., 2012). In the absence of neurophysiological investigation in this field with felines, we must be open to the possibility that either neural mechanism (if not both) is also involved in the lionesses’ perception of illusory motion.

We then asked what the benefits of this type of visual material are to lionesses’ behavior. First of all, the lionesses under investigation repeatedly interacted with the PVC sheets by biting them, holding them in the mouth, and dragging them around the enclosure, and they revisited them several times during the observation sessions. Thus, the PVC sheets stimulated the performance of species-specific behaviors related to hunting and prey subjugation (Kruuk and Turner, 1967; Schaller, 1972; Lindburg, 1988).

Considering the activity of the lionesses, the visual stimuli seemed to reduce the inactivity of Lubaya. Carnivores tend to be inactive in a controlled environment and in the presence of visitors (Shepherdson et al., 1993; Mellen et al., 1998), and zoo lions have been found to be particularly difficult in terms of increasing activity level (Powell, 1995). Thus, this finding seems to underlie a positive welfare implication, promoting active behavior in species that tend to be inactive in controlled environments. Moreover, when focusing on the time spent in individual behaviors, the visual stimuli seemed to influence the behavior of all subjects. In particular, they had some positive effects in the case of Kianga. Indeed, Kianga performed more self-grooming at baseline than in the RS period. Self-directed behavior, particularly self-grooming, is normal in cats because it is used to clean the fur and maintain insulation properties (Eckstein and Hart, 2000; Virga, 2005). However, this behavior might also indicate a stressful or conflict situation, highlighting possible welfare issues (Powell, 1995; Virga, 2005). Therefore, the decrease in self-grooming in Kianga during the RS period might underline a welfare improvement, although in both periods, Kianga exhibited low levels of self-grooming. The reduction in this behavior in the presence of the visual stimuli might indicate that the study lioness spent more time showing other relevant species-specific behaviors. In particular, this female showed more attentive behavior in the RS period than at baseline. The reported increase in attentive behavior might therefore indicate a positive welfare implication, because in the presence of the visual stimuli, Kianga became more vigilant, and reactive. This finding aligns with previous research on the effect of environmental enrichment in African lions, reporting that the presence of novel objects as enrichment devices increased activity and alertness in this species (Powell, 1995; Van Metter et al., 2008). Similar findings have been reported in other felids, such as black-footed cats (Wells and Egli, 2004) and tigers (Van Metter et al., 2008). On the other hand, in the RS period, Kianga performed more anticipatory behavior than at baseline. During the RS period, the zoo closing time was earlier than at baseline. Thus, it is possible that the lionesses expected to enter the indoor area, in which the daily amount of food was provided, and performed anticipatory behavior before the end of the data collection session. This behavior has been described as a potential welfare indicator (Ward et al., 2018), but it seems not to be related to the presence of the visual stimuli, as it is directed toward the indoor area of the enclosure during a specific period of the day.

Within individual behaviors, Lubaya exhibited more maintenance in the RS period than at baseline. This result might be linked to the decrease in inactive behavior observed in Lubaya during the RS period, suggesting that in the presence of the novel stimuli, this subject performed more species-specific behaviors.

Regarding Safia, we reported an increase in the interaction with the environmental enrichment stimuli and therefore the performance of species-specific behaviors such as play and hunting-related activities, suggesting a positive welfare implication for this lioness (Powell, 1995; Baker, 1997; Hosey et al., 2013).

The visual stimuli also impacted the social behaviors of Kianga and Lubaya, particularly affiliative behaviors. Indeed, Kianga exhibited significantly more affiliative behaviors in the RS period, whereas a trend toward significance (*p* = 0.05) was reported for Lubaya. Thus, the novel stimuli seemed to improve positive social interactions among lionesses, as previously reported in other studies describing the benefits of environmental enrichment for African lions in zoos (Powell, 1995; Baker, 1997). Moreover, Kianga performed more interspecific social behavior, intended as attention toward humans such as visitors and zookeepers, at baseline than in the RS period. Zookeepers and other human factors have been found to be major determinants of animal welfare and could be, in some cases, stressful for the animals, leading to negative reactions toward the public as well as to the development of abnormal behaviors (Hosey, 2000, 2008, 2013; Davey, 2007; Fernandez et al., 2009; Cole and Fraser, 2018). The reduction of interspecific social behavior of Kianga seems therefore to be positive for the welfare of the subject, as the decrease in time spent interacting with humans might indicate an increase in the performance of other desirable species-specific behaviors, underlining improvements in the animal’s psychological well-being (Cole and Fraser, 2018).

**FIGURE 5:**
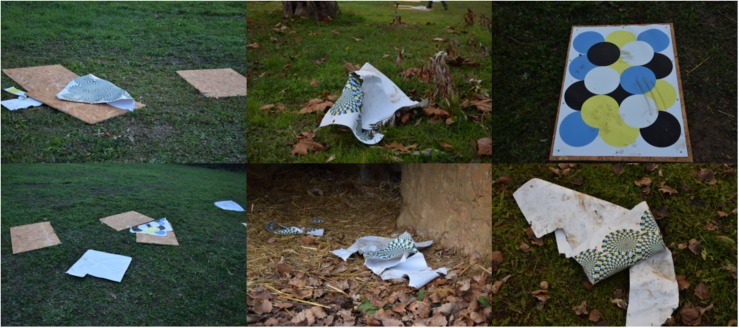
Example of remains of the stimuli after an experimental session.

The materials used in the current study are convenient, because the PVC sheets can be printed quickly and cheaply in any print shop. However, to avoid competition between subjects, more than one stimulus per subject is needed because they can generate great interest in the subjects. Moreover, although resistant to water and bad weather conditions, the PVC sheets can easily and potentially be destroyed by the lions and are not always long-lasting or reusable (Figure 5). Instead of PVC sheets, environmentally friendly cloths can be used. Based on our experience, the visual stimuli can be provided to the animals on non-consecutive days to be more efficient (e.g., once a week) and can be left in the lions’ enclosure for the whole day, because each individual of the current study played with the PVC sheets and their remains at more times during the day. We suggest that visual stimuli such as motion illusions could be included in the environmental enrichment schedule of lions and possibly other carnivore species in zoos but should be alternated with other types of stimulations (e.g., olfactory, manipulative, and food-related devices) to promote the widest array of species-specific behaviors related to positive welfare.

We are aware that the small sample size – unfortunately, a methodological flaw shared with many studies involving zoo animals (Perdue et al., 2012; Vonk and Beran, 2012; Fuller et al., 2018) – prevents drawing any firm conclusions on the real preference of lionesses for interacting with the RS illusion and the potential impact of such a stimulation in their environment. However, the data included in this study are promising and call for a larger investigation on the use of motion illusions as visual environmental enrichment in natural parks. The illusory pattern seems to have been explored more than other stimuli with a similar configuration, where the perception of motion is absent. Overall, this type of material seems to provide some benefits in the expression of species-specific behaviors of big cats. We hope that in the near future, this hypothesis could be tested on a larger range of individuals and species.

## Data Availability Statement

The datasets generated for this study are available on request to the corresponding author.

## Ethics Statement

Ethical review and approval was not required for the animal study because the study was carried out through the behavioral observation of the lionesses, using non-invasive techniques. The study procedure was in accordance with the EU Directive 2010/63/EU and the Italian legislative decree 26/2014 for Animal Research. No special permission to use animals in the current ethological non-invasive study is required, as zoological gardens in Italy are expected to carry out behavioral observations of the individuals in their care, in order to guarantee the animal welfare (D. Lgs.73/2005).

## Author Contributions

MM, CS, and CA developed the study concept. BR, AR, and GO collected the data. BR, AR, CS, and CA analyzed the data. BR, MM, CS, and CA wrote the manuscript.

## Conflict of Interest

BR is employed by the Parco Natura Viva as researcher in the Research and Conservation Department. GO is employed by the Parco Natura Viva as General Curator assistant. CS is employed by the Parco Natura Viva as head of the Research and Conservation Department.

The remaining authors declare that the research was conducted in the absence of any commercial or financial relationships that could be construed as a potential conflict of interest.
